# Shifting paradigms and creating space for Indigenous leadership in biosecurity management and decision‐making

**DOI:** 10.1111/cobi.14399

**Published:** 2024-11-25

**Authors:** Tracey Godfery, John Kean, Daniel Hikuroa, Andrew Robinson, Nari Williams

**Affiliations:** ^1^ School of Biological Sciences Waipapa Taumata Rau – University of Auckland Auckland New Zealand; ^2^ AgResearch Limited Ruakura Research Centre Hamilton New Zealand; ^3^ Faculty of Arts, Māori Studies Te Wānanga o Waipapa, Waipapa Taumata Rau – University of Auckland Auckland New Zealand; ^4^ School of BioSciences University of Melbourne Melbourne Victoria Australia; ^5^ School of Mathematics and Statistics University of Melbourne Melbourne Victoria Australia; ^6^ Plant and Food Research Havelock North New Zealand; ^7^ George Mason Centre for the Natural Environment, School of Biological Sciences Waipapa Taumata Rau – University of Auckland Auckland New Zealand

**Keywords:** biosecurity management, contrasting worldviews, Indigenous paradigms, nature relationships, gestión de la bioseguridad, paradigmas indígenas, relaciones con la naturaleza, visiones contrastantes, 自然关系, 土著人民的范式, 截然不同的世界观, 生物安全管理

## Abstract

In New Zealand, awareness regarding protection, enhancement, and regeneration of landscapes and biodiversity is growing as the relationship between functioning and diverse ecosystems and society's health is acknowledged. This relationship is especially important for Indigenous people, who hold strong genealogical and familial ties with nature. Significant biodiversity loss from anthropogenic factors is exacerbated by climate change, ecosystem degradation, and invasive species. Invasive species and other biological threats, such as native pathogens, are concerning for Māori communities, who hold cultural responsibilities to care for nature. Despite acknowledgment of the value of Indigenous perspectives in environmental management in New Zealand and globally, Indigenous participation still largely occurs within Western non‐Indigenous paradigms. We highlight the *value of* Indigenous participation in biosecurity management and propose a shift from Western‐based paradigms to paradigms that reflect Indigenous worldviews and relationships with place. Recognizing and including the value of Indigenous participation elevates Indigenous voices to the level of decision‐making and leadership in the management of Indigenous lands. Given the genealogical relationships that Māori hold with the natural world and the intertwining of their health and well‐being with that of place (land) and nature, biosecurity threats to native species and ecosystems also pose serious risks to community well‐being. A holistic biosecurity approach is needed that encompasses cultural, social, economic, and environmental factors at multiple scales. We examined the New Zealand biosecurity context relative to biological threats to native plants and ecosystems and proposed a paradigm shift toward Indigenous place‐based biosecurity management. Biosecurity science and science‐based tools remain an important component, underscoring the complementary aspects of science and (Indigenous) culture.

## Abstract (es)

Cambios de paradigmas y creación de espacios para el liderazgo indígena en la gestión y decisiones de bioseguridad

**Resumen:**

En Nueva Zelanda hay un aumento en la conciencia sobre la protección, mejora y regeneración de los paisajes y la biodiversidad a medida que se reconoce la relación entre el funcionamiento y la diversidad de los ecosistemas y la salud de la sociedad. Esta relación es especialmente importante para los pueblos indígenas, que mantienen fuertes lazos genealógicos y familiares con la naturaleza. La pérdida importante de biodiversidad por factores antropogénicos está agravada por el cambio climático, la degradación de los ecosistemas y las especies invasoras. Las especies invasoras y otras amenazas biológicas, como los patógenos autóctonos, preocupan a las comunidades maoríes, que tienen la responsabilidad cultural de cuidar de la naturaleza. A pesar de que en Nueva Zelanda y en el resto del mundo se reconoce el valor de las perspectivas indígenas en la gestión del medio ambiente, la participación de los indígenas sigue teniendo lugar en gran medida dentro de paradigmas occidentales no indígenas. Resaltamos el valor de la participación indígena en la gestión de la bioseguridad y proponemos un cambio de paradigmas occidentales a paradigmas que reflejen las cosmovisiones indígenas y las relaciones con el lugar. El reconocimiento y la inclusión del valor de la participación indígena eleva las voces indígenas al nivel de toma de decisiones y liderazgo en la gestión de las tierras indígenas. Dadas las relaciones genealógicas que los maoríes mantienen con el mundo natural y la conexión entre su salud y bienestar con los del lugar (la tierra) y la naturaleza, las amenazas a la bioseguridad de las especies y ecosistemas autóctonos también suponen un grave riesgo para el bienestar de la comunidad. Es necesario un enfoque holístico de la bioseguridad que abarque factores culturales, sociales, económicos y medioambientales a múltiples escalas. Analizamos el contexto de la bioseguridad en Nueva Zelanda en relación con las amenazas biológicas a las plantas y ecosistemas autóctonos y proponemos un cambio de paradigma hacia una gestión de la bioseguridad basada en el lugar indígena. La ciencia de la bioseguridad y las herramientas basadas en la ciencia siguen siendo un componente importante, que subraya los aspectos complementarios de la ciencia y la cultura (indígena).

## 转变范式并为原住民领导力创造空间 生物安全管理和决策

【摘要】 在新西兰, 随着人们逐渐认识到生态系统的正常运作和多样性与社会健康之间的关系, 保护、改善和恢复景观和生物多样性的意识也在不断提高。这种关系对土著人民尤为重要, 因为他们与自然之间有着紧密的系谱和家族联系。气候变化、生态系统退化和物种入侵加剧了人为因素造成的生物多样性严重丧失。毛利人社区对物种入侵和包括本地病原体在内的其他生物威胁感到担忧, 因为他们承担着关爱自然的文化责任。尽管新西兰和全球都承认土著人民的观点在环境管理中的价值, 但土著人民的参与在很大程度上仍受限于西方非土著的范式。本研究强调了土著人民参与生物安全管理的价值, 并建议从西方范式向反映土著人民世界观和和地方关系的范式转变。承认土著人民参与的价值并将其纳入现有体系, 有助于将土著人民的声音提升到土著土地管理的决策和领导层面。考虑到毛利人与自然世界的系谱关系, 以及他们的健康和福祉与地方(土地)和自然的交织关系, 生物安全对原生物种及生态系统的威胁也会对社区福祉构成严重风险。因此, 需要开发一种在多尺度上涵盖文化、社会、经济和环境因素的综合性生物安全方法。我们分析了新西兰生物安全背景下本地植物和生态系统面临的生物威胁, 并提出了向基于土著土地的生物安全管理范式转变的建议。生物安全科学和基于科学的工具仍是这一范式的重要组成部分, 这也强调了科学与(土著)文化的互补性。**【翻译:胡怡思;审校:聂永刚】**

## INTRODUCTION

In New Zealand, awareness regarding protection, enhancement, and regeneration of landscapes and biodiversity is growing as the relationship between functioning and diverse ecosystems and society's health is acknowledged (Department of Conservation, 2020). This relationship is especially important for Indigenous people, who hold strong familial ties with nature (Adese, 2014; Herman, 2008; Hikuroa et al., 2018; Lyver et al., 2019). For New Zealand's Indigenous people (Māori), environmental care that is premised on respect and reciprocity underpins interactions between people and nature, as evidenced in sayings such as “If we look after the land, the land will look after us” (Godfery & Kelly, 2018; Zinsstag et al., 2011).

Human settlement in New Zealand brought unparalleled change to the natural environment (McCulloch, 1988), with forest cover reduced from 85% pre‐human settlement to 24.8% by 2005 (Warne, 2014; Wilmshurst, 2007). In the wake of this change, McCulloch (1988) highlighted the contrasting views of humankind as part of nature as opposed to apart from nature, emphasizing humans as a product of nature (and therefore part of it) but with the ability to create extensive change. Despite New Zealand's anthropogenic‐induced deforestation, the perception of humans as part of nature strongly aligns with Māori worldviews and supports the protection, enhancement, and regeneration of New Zealand's natural environment.

Significant anthropogenic biodiversity loss is exacerbated by climate change, ecosystem degradation, and invasive species. New Zealand's relative isolation and cultural environment, however, allow the country's biosecurity to “[extend] to the problem of invasive species and preserving the integrity and persistence of particular ecologies, often related to…national identity” (Braun, 2013, p. 45).

Even though Indigenous perspectives in environmental management are recognized in New Zealand and globally (Barbour & Schlesinger, 2012; Buell et al., 2020; CBD, 1992, 2022; CBD Secretariat, 2000; Reed et al., 2021), Indigenous participation still largely occurs within Western non‐Indigenous paradigms. Consequently, full and equal Indigenous involvement is often lacking (Buell et al., 2020; Kuru et al., 2021; Lyver et al., 2019), tokenistic, or culturally appropriative. Indigenous knowledge and wisdom should not be used to achieve outcomes that predominantly serve non‐Indigenous priorities at the expense of Indigenous priorities.

We examined the difference between Western and Indigenous worldviews of nature and between *valuing* (often resulting in paternalism) and the *value of* (supports authentic engagement and collaboration) Indigenous participation. We explored New Zealand's biosecurity system and pathogen threats to native trees and a proposed shift toward Indigenous place‐based biosecurity management in this context. Reflecting a complementary approach that builds on the strengths of New Zealand's current biosecurity system and Indigenous culture, our model has implications for Indigenous involvement in environmental and conservation contexts worldwide. Table 1 summarizes key elements of the current and proposed approaches.

**TABLE 1:** Comparison of key elements of New Zealand's current biosecurity management and a proposed paradigm shift.

Current paradigm (non‐Indigenous)	Future paradigm (Indigenous)
Top‐down management approach Hierarchical nature view of human dominance—humankind apart from nature Western science‐based management, assisted by Indigenous knowledge Valuing^a^ Indigenous participation Disparate elements of Indigenous knowledge and culture Knowledge and wisdom separated Indigenous participation in decision‐making uncertain National biosecurity approach—single threat, to multiple locations Risk categories isolated; Indigenous perspectives confined to cultural values Strong focus on primary industries and the national economy	Bottom‐up community‐based approach Interconnected and relational nature view (nonhierarchical)—humankind as part of nature Indigenous place‐based management, assisted by science and technology The value of^a^ Indigenous participation Holistic knowledge incorporated as part of worldview Knowledge and wisdom are intertwined Indigenous leadership and autonomy in decision‐making Localized biosecurity approach—multiple threats, to single location Risk categories connected; well‐being influenced by economic, environmental, cultural, and social factors Strong focus on sustainable livelihoods and communities

^a^ Valuing shows an appreciation of Indigenous participation but within non‐Indigenous paradigms, whereas the value of Indigenous participation more appropriately allows for Indigenous involvement in decision‐making and within Indigenous paradigms.

## CONTRASTING WORLDVIEWS

Nature‐based genealogies are common in Indigenous societies. For example, *whakapapa* (genealogy) underpins the Māori worldview and includes physical and metaphysical elements of the natural world (Marsden, 2003; Marsden & Henare, 2003; Morgan, 2004). Therefore, for Māori, the health and wellness of people is intimately bound to the health of nature (Lambert et al., 2018; Lyver et al., 2019; Marsden, 2003). The strength of this connection and mutual wellness is reflected in the proverb “*E rere kau mai te awa nui mai i Te Kāhui Maunga ki Tangaroa*; *ko au te awa, ko te awa ko au*” (“The great river flows from the Mountains to the Sea; I am the river, the river is me.”) (Rangiwaiata Rangitihi Tahuparae, as cited in Wilson [2010]). Similarly, the genealogical relationship between New Zealand kauri trees and the people of Te Roroa is expressed in “*Ko te kauri, ko au, Ko au, ko te kauri*” (“I am the kauri, the kauri is me.”) (Ngakuru et al., 2010, p. 16).

The Taku River Tlingit First Nation (in North America) similarly acknowledges, “It is the land from which we came that connects all life” and “Our land looks after us and we look after our land” (Taku River Tlingit First Nation Constitution Act, 1993). Indigenous Andean people view human existence as contingent on harmony with nature, acknowledging everything has life and is part of Pachamama (Mother Earth) (Sólon, 2018). Evidence of the strong relationships between Indigenous peoples and their lands is also seen in the legal recognition of nature: Rights for Nature in Ecuador (Berros, 2015), Law of the Rights of Mother Earth in Bolivia (Añaños Bedriñana et al., 2020), and the legal personhood of the Whanganui River and Te Urewera Ranges in New Zealand (Geddis & Ruru., 2019; Hutchison, 2014).

These familial relationships with nature are encapsulated in references to Earth as “…a living organism… creating and supplying a web of support systems for all [life]…” (Marsden, 2003, p. 45). Māori genealogical relationships with Earth and nature are highlighted in *whenua*, which means land or natural earth and human placenta. This dual meaning affirms the Māori belief that humans “are born out of the womb” of Earth (Marsden, 2003, p. 45). Such genealogical connections with nature imbue reciprocity and respect that contribute to upholding the interconnectedness and balance of nature.

Notwithstanding increasing appreciation of nature, capitalism supports the notion of land as property to be exploited for profit. For most Indigenous peoples, however, land as property or private good is an alien concept; reciprocal obligation and duty of care are embedded in land and nature relations (Frame, 1999; Marsden & Henare, 2003; Te Awa Tupua Act 2017, 2017; Te Urewera Act 2014, 2014). This worldview contrasts sharply with Western‐based views of land as property and nature as resource (Mutu, 2022). These different perceptions and understandings create oppositional paradigms for managing the natural world (Western paradigm) or living in relation with it (Indigenous paradigm).

Hardin's (1968) tragedy of the commons theory assumes commons users will eventually destroy the commons on which they depend. This fits Western constructs but fails to recognize the communal systems of Indigenous communities. When land and nature are integral to community relationships, communal benefit is reciprocated in the collective relationship. Hardin (1998) later noted that managed commons may succeed or fail (whether managed communally or as private good), whereas unmanaged commons are doomed to fail because exploitation inevitably exceeds ecological limits. This modification better supports Indigenous systems of communal management and offers an alternative to the view of commons as private good.

Although Indigenous cultures and beliefs are diverse, their common worldviews based on polytheistic, animistic beliefs create a shared ethos of reciprocity and respect for nature. Generally, the Western view of nature is that humankind is separate and dominant and that land and nature are resources for human benefit.

Integrating Western and Indigenous worldviews in environmental management, which some warn against, may result in adversarial paradigms. Tau (2001) proposed Māori knowledge (*mātauranga*) be viewed separately to avoid fitting knowledge with a different foundational base into another (Western) knowledge system. Different knowledges can be applied in parallel, but whether mātauranga Māori can “be applied in conjunction with other systems” is unclear (Durie, 2005, p. 138).

Ahenakew (2016, p. 323) referred to “grafting” or the “transplanting ways of knowing and being from a context where they emerge naturally [Indigenous] to a context where they are artificially implanted [non‐Indigenous].” Ahenakew (2016, p. 323) also highlighted conditional forms of integration that support dominant ways of thinking by presenting them as “benevolent and inclusive.” This transplanting and benevolent and inclusive integration highlights the different perceptions that arise from Indigenous and non‐Indigenous groups. Careful attention to ensure Indigenous knowledge is applied appropriately and within Indigenous paradigms is essential, including attention to contrasting worldviews and how these influence aspirations, knowledge application, and outcomes.

## VALUING AND THE VALUE OF INDIGENOUS PARTICIPATION

If included, Indigenous perspectives are often incorporated in Western‐based frameworks and serve a Western paradigm (Ahenakew, 2016; Buell et al., 2020; Fletcher et al., 2021; Kuru et al., 2021; Reed et al., 2021). Such participation demonstrates appreciation for or valuing the Indigenous contribution; however, Indigenous participation in decision‐making within their (Indigenous) paradigm of origin more appropriately supports the value of Indigenous participation.

Inclusive and Māori‐led research has developed approaches that elevate Māori cultural perspectives and ensure meaningful participation in New Zealand's environmental management and decision‐making processes (Lyver et al., 2019; Morgan, 2004; Taura et al., 2017; Tipa & Nelson, 2008; Tipa & Teirney, 2006). Models underpinned by an extensive Māori knowledge base and worldview offer a foundation for ongoing engagement and work in this space—for example, using biocultural approaches to enhance local decision‐making and strengthen *tuakana‐teina* relationships with nature (Lyver et al., 2019). Tuakana‐teina denotes relationships between older and younger siblings (Keane, 2011) and is characterized by reciprocal learning (Lyver et al., 2019). Tuakana‐teina nature relationships acknowledge nature as an elder entity, provisioning life‐sustaining benefits that humans have a reciprocal responsibility to return through care. The role of Indigenous communities in conservation therefore emphasizes the value of inherent nature–human relationships.

In the Canadian Great Lakes region, Buell et al. (2020) applied an alternative approach to ecological risk assessment in the territory of the Saugeen Ojibway Nation. Research decisions reflected Indigenous perspectives, knowledge, and concerns, and Western science and traditional ecological knowledge (TEK) were used to conduct scientific risk assessments. Despite, for example, the meaningful and collaborative partnerships and embedding of TEK in risk assessment, the authors cautioned against universal application, reiterating the unique perspectives, relationships, and values held by different Indigenous communities.

Indigenous inclusion may, however, still replicate Western‐based management. In Australia's Kimberley Region, 27 of 35 Aboriginal approaches to weed control used government lists of species, and only 2 programs met cultural aspirations (Bach et al., 2019). However, where locations were managed according to geographies of healthy country and incorporated culturally specific landscape and vegetation management, communities experienced more positive outcomes.

Scope remains for Indigenous participation in environmental management to extend to decision‐making. Fletcher et al. (2021) note, “Indigenous and local peoples across the globe have long advocated, that their voices, concerns, and needs…take precedence in …conservation governance arrangements that involve their ancestral territories and embrace multifunctional landscapes” (Fletcher et al., 2021, p. 5). Prioritizing Indigenous voices ensures Indigenous perspectives and knowledge are applied appropriately, benefiting communities and their respective lands.

Valuing Indigenous participation by merely integrating Indigenous knowledge and culture in Western‐based approaches invariably leads to imposing Western values and practices over Indigenous landscapes. Such imposition separates traditional knowledge from the people, place, and culture to which it belongs. Indigenous knowledge that is spatially specific may be inappropriately applied universally. Furthermore, traditional knowledge may be separated from traditional wisdom. For Māori “[k]nowledge is a thing of the head, an accumulation of facts[, whereas] [w]isdom is a thing of the heart. It has its own thought processes. It is there that knowledge is integrated for this is the centre of one's being” (Marsden & Henare, 2003, p. 59). Interpretation of Indigenous knowledge and its application in environmental settings require understanding that encompasses elements beyond objective science‐based knowledge and includes the wisdom that guides and informs Indigenous knowledge. Creating space for this holistic understanding appropriately acknowledges the value of Indigenous participation, providing an equitable pathway to partnership and Indigenous decision‐making.

## NEW ZEALAND'S BIOSECURITY

New Zealand's biosecurity system is managed by the Ministry for Primary Industries (MPI) and delivered through Biosecurity New Zealand. The system engages with Māori communities, affected stakeholders, and the wider public. Engagement with Māori reflects system strengths in valuing Indigenous participation; however, an opportunity exists to shift this valuing to a paradigm that instead recognizes and advances the value of Indigenous participation to support Māori communities to lead and drive biosecurity response and management.

Geographic isolation and the country's sea border provide considerable protection from biosecurity threats; the country is free of many pests and diseases found elsewhere (Biosecurity New Zealand, 2024). However, the global movement of people and goods presents problems for keeping non‐native organisms out, and once arrived, localized transfer complicates eradication and contributes to reinfestations (Robinson & McNeill, 2022). Plant pathogens in particular are a “pernicious” threat to native New Zealand forests (Warne, 2014). A robust biosecurity system is thus crucial to minimizing potential risks.

Underpinned by risk assessment and risk management, New Zealand's biosecurity system comprises 3 interconnected levels—pre‐border, at‐border, and post‐border protection. These levels are supported by invasion biology research on past incursions, species’ biology of organisms yet to arrive, and biophysical factors influencing species introduction and spread. Risk mitigation at each level helps reduce incursion risk. Increasing community awareness and participation in biosecurity is also vital to its success, as evidenced by New Zealand's citizen reporting of myrtle rust (*Austropuccinia psidii*). Citizen scientists (*n* = 1304) using iNaturalist recorded 3363 observations from November 2017 to September 2024 (iNaturalist, 2024).

Biosecurity New Zealand's Kauri Protection program (Tiakina Kauri) demonstrates MPI's commitment to collaboration with Māori. The program's partnership supports Māori involvement through a Tangata Whenua Roopu (Māori representation) and representation of Te Iwi o Te Roroa, the tribe whose Treaty settlement with the Crown (represented by the New Zealand Government) includes cultural redress and protections in relation to a significant kauri forest at Waipoua (New Zealand Government, 2009).

Similarly, the collaborative Beyond Myrtle Rust program (funded by the Ministry of Business, Innovation and Employment) includes Māori research perspectives and knowledge. The research examines multiple facets of the disease and incorporates mātauranga Māori and capacity building and strategy development to facilitate Māori leadership (Manaaki Whenua Landcare Research, 2022).

New Zealand's biosecurity system is demonstrably inclusive, encouraging community and citizen participation and employing up‐to‐date science in risk assessment and biosecurity response and management. Although the system includes Māori communities and other groups, it primarily serves industry and is weighted toward economic risk and impacts. This weighting results in deficiencies in other areas of the system.

### Gaps in an otherwise inclusive system

Gaps in New Zealand's biosecurity system were identified by Dyck and Hickling (2021) and Newfield and Reed (2021). Limitations identified by these authors included limited consideration and capability in environmental, social, and cultural risk and ability to predict impacts of non‐native pathogens. Attention to cultural and social values and impacts and an absence of tools for considering these values within the system were also lacking (Newfield & Reed, 2021). This is supported by Teulon et al. (2015), who found significant attention to economic and environmental impacts of myrtle rust and little attention to cultural impacts.

New Zealand's science capability and capacity to address pathogen threats to plants are also lacking (Dyck & Hickling, 2021).

[T]here is little investment in protecting native forests from pest and pathogen incursions. Recent highly damaging incursions such as kauri dieback and myrtle rust [show]… native species may be less resilient than was once thought. Sophisticated science and technology is routinely applied to protect New Zealand's crops, orchards and plantation forests; native forests must be included in future (Dyck, 2021).

The reports cited here acknowledge positive aspects of New Zealand's biosecurity system; however, clear gaps are also identified. The focus on primary industries and related economic risk is disproportionate, and increased consideration of other risk categories and native ecosystems is needed. The need for significant attention to biosecurity threats to New Zealand native forests is pressing and is especially crucial for Māori, whose strength of connection with their naturescapes means biosecurity threats to native trees and forests also threaten the well‐being of related tribes.

### Plant pathogen threats to the natural landscape

Plant pathogens *Phytophthora agathidicida* and *Austropuccinia psidii* threaten some of New Zealand's most treasured tree species, New Zealand kauri (*Agathis australis*) and native Myrtaceae species, respectively, including pōhutukawa (*Metrosideros excelsa*), a species of cultural and ecological importance. These and other threats concern Māori communities, who maintain cultural responsibilities to care for nature as *kaitiaki* (caregivers) (Black et al., 2019; Hill et al., 2021; Lambert et al., 2018; Teulon et al., 2015; Tora, 2019). Kauri, an iconic and endemic New Zealand tree, is revered by northern tribes. Its metaphysical significance is evident in its inclusion in creation narratives and its chiefly status (Orwin, 2007).

**FIGURE 1:**
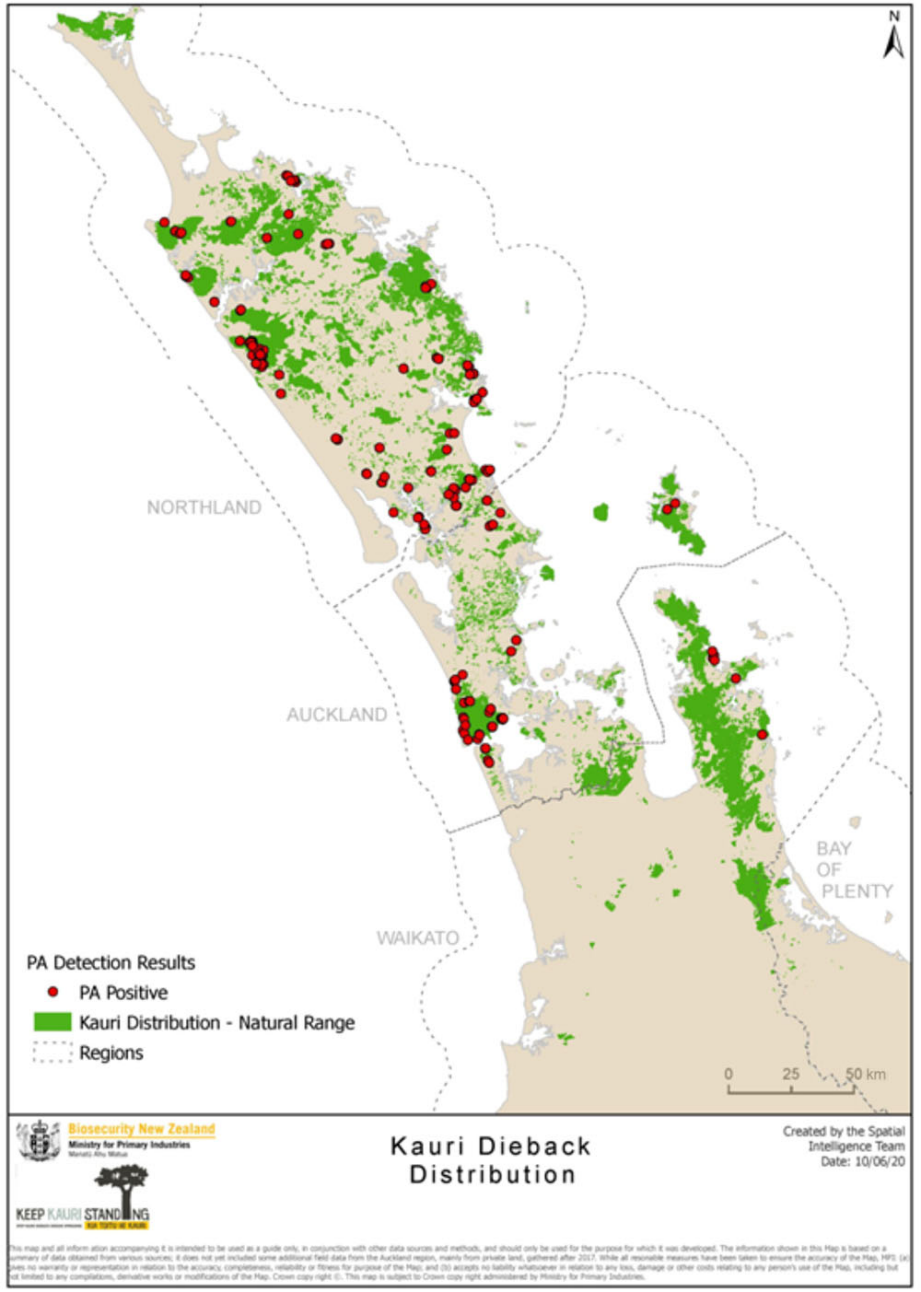
Distribution of kauri and kauri dieback disease. *Source*: Ministry for Primary Industries, licensed for reuse under the Creative Commons Attribution 4.0 International license.

Kauri is the only member of the Araucariaceae family, the largest native tree in New Zealand, and one of the world's largest trees by wood volume (Dawson & Lucas, 2012). Individual trees occur throughout New Zealand, though kauri forests occur predominantly in Northland, Auckland, and Coromandel. Latitude and elevation restrict species natural distribution to north of 38° S and 600 m asl (Orwin, 2007). In 2015, kauri was classified as threatened due to the spread of *P. agathidicida* (Department of Conservation, 2018), now present throughout kauri's native range (Figure 1).

The myrtle pōhutukawa is also an iconic New Zealand tree, occurring throughout the North Island south to Poverty Bay in the east and northern Taranaki in the west (Dawson & Lucas, 2012). Pōhutukawa are salt tolerant and critically important in coastal ecosystems, reducing coastal erosion and sediment influx to waterways (Smith et al., 2020).

Certain individual pōhutukawa are revered by Māori for their cultural and spiritual significance (Teulon et al., 2015). Te Rēinga, an 800‐year‐old pōhutukawa, at the northwestern tip of New Zealand, represents the sacred portal through which spirits embark on their homeland journey (Ministry for Culture & Heritage, 2016). Te Papa o Karewa and Tangi te Korowhiti are pōhutukawa immensely important to descendants of the Tainui *waka* (canoe). The area between the trees provided the waka's landing place, and Tangi te Korowhiti provided a mooring for the waka (Historic Places Trust, 2009). Te Waha o Rerekohu at Te Araroa is believed to be New Zealand's largest pōhutukawa (38.5 m branch span) (Teulon et al., 2015). Cared for by local *hapū* (subtribe) Te Whānau a Hinerupe, Te Waha o Rerekohu has lived among families for 24 generations (Morton, 2023). Te Waha o Rerekohu is infected with myrtle rust.

**FIGURE 2:**
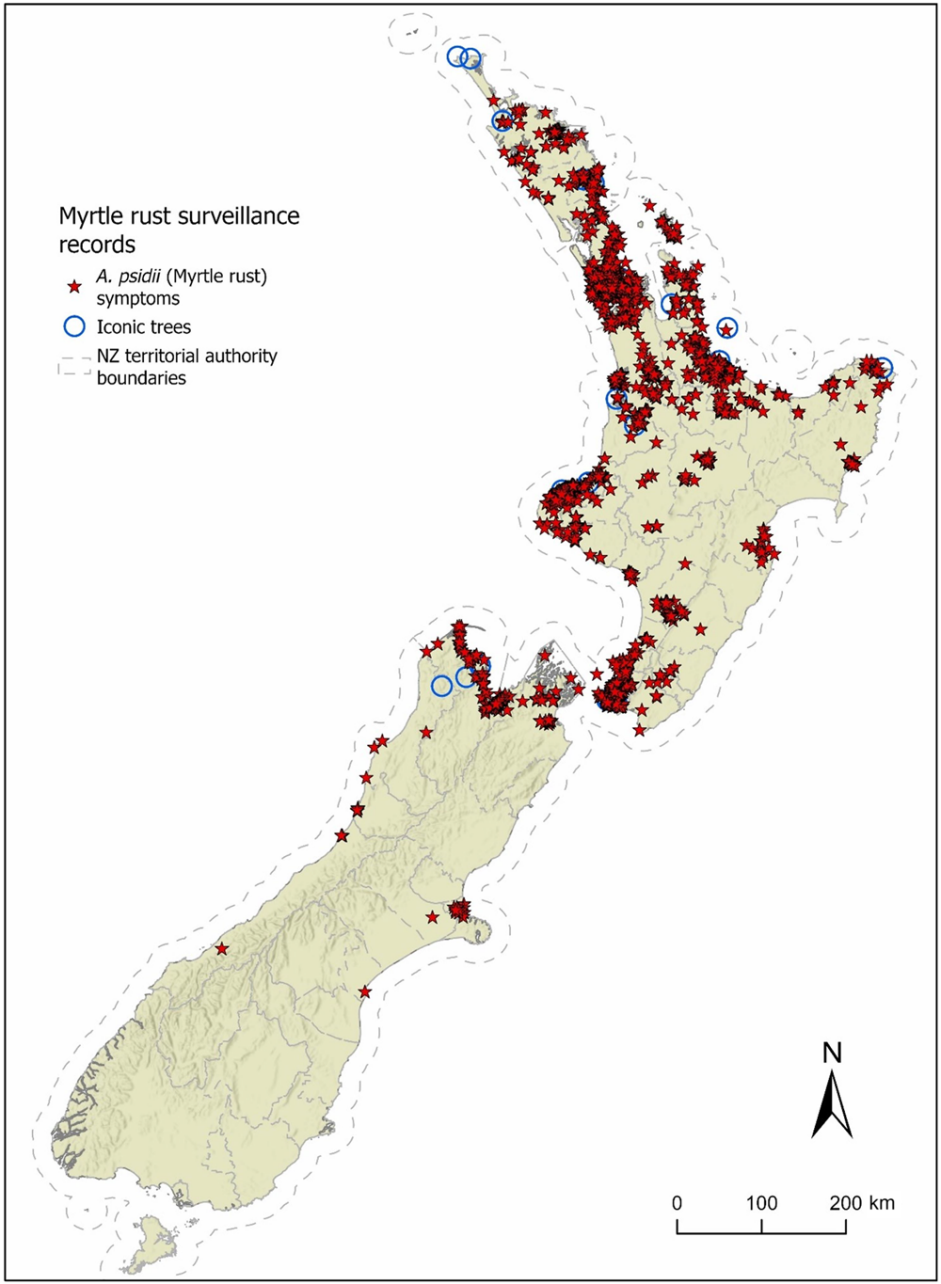
Known distribution of myrtle rust in New Zealand as at November 2023 (Campbell et al., 2021). *Source*: Data from the myrtle rust surveillance library were compiled from the Ministry for Primary Industries (MPI), the Department of Conservation (DOC), the New Zealand Institute for Plant and Food Research Ltd, Botanic Gardens, and iNaturalist (Myrtle Rust Reporter App, https://www.inaturalist.org/projects/myrtle‐rust‐reporter).

There are almost 100 Myrtaceae (species, subspecies, hybrids, cultivars) in New Zealand (Dawson et al., 2019); 29 are native, and 28 of these are endemic (New Zealand Plant Conservation Network, 2022). The impact of myrtle rust on native Myrtaceae and the potential future threat of *Ceratocystis fimbriata* to *Metrosideros* have resulted in changes in threat classification of all New Zealand native Myrtaceae (de Lange et al., 2018). Ten species are nationally critical, one species is nationally endangered, 17 species are nationally vulnerable, and one species, mānuka (*Leptospermum scoparium* var. *scoparium*), is declining (New Zealand Plant Conservation Network, 2022). Myrtle rust is widely distributed in New Zealand (Figure 2).

Biosecurity New Zealand's long‐term management programs include *P. agathidicida*, although management of *A.psidii* is more difficult, in part due to the pathogen's aerial dispersal.   Further threats to native trees and forests of not‐yet‐arrived pathogens are also identified, including *Phytophthora ramorum*, which has killed millions of oaks and tanoaks in the western United States, and *Ceratocystis fimbriata*, a fungus that could threaten pōhutukawa and rātā. On the island of Hawai'i, native ‘ōhi'a (*Metrosideros polymorpha*) have been devastated by rapid ‘ōhi'a death, originally attributed to *C. fimbriata*; the disease is now known to be caused by *Ceratocystis lukuohia* and *Ceratocystis huliohia* (Barnes et al., 2018). New Zealand's pōhutukawa and rātā trees are closely related to the ‘ōhi'a.

Other pathogen threats include those of native origin. The phytoplasma bacterium *Candidatus* Phytoplasma australiense is a native pathogen and causes 4 plant diseases, 3 of which affect native species. Native pathogens therefore exacerbate risks posed by potential non‐native pathogens.

Current threats, future threats, and emerging native pathogens could have immense impact on cultural, environmental, social, and economic well‐being. These possibly compounding threats to native trees and forests emphasize the need for an inclusive biosecurity system that incorporates risks and impacts to local communities and recognizes their respective values and perspectives in totality—that is cultural, environmental, and social, as well as economic factors.

## SHIFTING BIOSECURITY MANAGEMENT TOWARD INDIGENOUS PARADIGMS

Biosecurity threats, especially plant pathogens, pose serious risks to native species and ecosystems. Despite geographic isolation and a robust biosecurity system affording some protection, combined biological threats and exacerbating anthropogenic pressures emphasize possible wide‐ranging risks and impacts. Thus, a holistic approach encompassing cultural, social, economic, and environmental factors at multiple scales—from species to ecosystems and across tribal landscapes—is needed. For Māori, a holistic biosecurity approach recognizes their enduring relationships with place.

New Zealand's biosecurity system, although implemented nationally, focuses on species directly affected by identified threats. Response and management are applied at pre‐border, border, and post‐border levels; post‐border biosecurity has a single‐threat, multiple‐location focus. This approach is appropriate for biosecurity threats to primary industries, for which biological threats affect monocultural crops grown in a defined geographic or climatic area. For example, a biological threat specifically affecting a horticultural crop has fewer variables to consider than one affecting the wider landscape. Influencing abiotic and biotic factors are more predictable, and generally standard growth conditions exist, making these less uncertain than more diverse natural environments. Typically, greater knowledge of relevant biosecurity science in the respective industry further reduces uncertainty.

However, a single‐threat, multiple‐location lens may not be as appropriate for threats to native species and ecosystems, for which localized biosecurity may better address compounding biological threats by considering discrete species effects; varying species tolerance or resilience relative to other locations; distinct ecosystem, climatic, or landscape factors; site‐specific anthropogenic pressures; and different perspectives and relationships communities maintain with place. Because biosecurity threats to native species and ecosystems may have different impacts and expression across sites, a single‐threat, multiple‐location approach seems antagonistic to full consideration of pathogen–host–environment relationships and the importance of this dynamic across diverse sites.

Another shortcoming of the single‐threat, multiple‐location approach is that it may not consider interactions between invasive taxa, which may be substitutive (new invader replaces a previous one, causing minimal additional damages), additive (new invader causes additional damages), or multiplicative (invader facilitates later invasions by weakening a host or ecosystem).

A threat, such as myrtle rust, that affects multiple species inhabiting different ecosystems over a wide‐ranging geographic area appears more difficult to address with a single‐threat, multiple‐location approach. The discovery of myrtle rust affecting Te Waha o Rerekohu occurred following humid weather in the East Cape and Cape Runaway areas of New Zealand's North Island. This area has already lost 3 native myrtles (ramarama [*Lophomyrtus bullata*], rōhutu [*Lophomyrtus obcordata*], akakura [*Metrosideros colensoi*]) since 2018, when the disease was first discovered in the area (Morton, 2023). Significant community and collaborative efforts in the fight against myrtle rust have occurred in this region (Biological Heritage NZ, 2020). This effort may have been better served by a place‐based approach that supports a direct focus on specific local conditions.

The complexity of ecosystem and species interrelationships and environmental variables across sites may increase uncertainty at a single location (Barry & Elith, 2006). However, across multiple locations, diverse influencing factors further increase uncertainty, potentially hindering successful biosecurity outcomes across sites. To increase controllability and lower uncertainty in socioecological systems, decision‐making factors to include are decision makers maintaining exclusive authority regarding the management of places or resources and stakeholders holding common aspirations for management outcomes (Rounsevell et al., 2021). With a nationwide focus (comprising multiple locations), uncertainty may therefore be higher from an ecological perspective, if requisite ecosystem and species knowledge is lacking across sites, and from socioecological perspectives when diverse stakeholders hold different aspirations and relationships with place.

**FIGURE 3:**
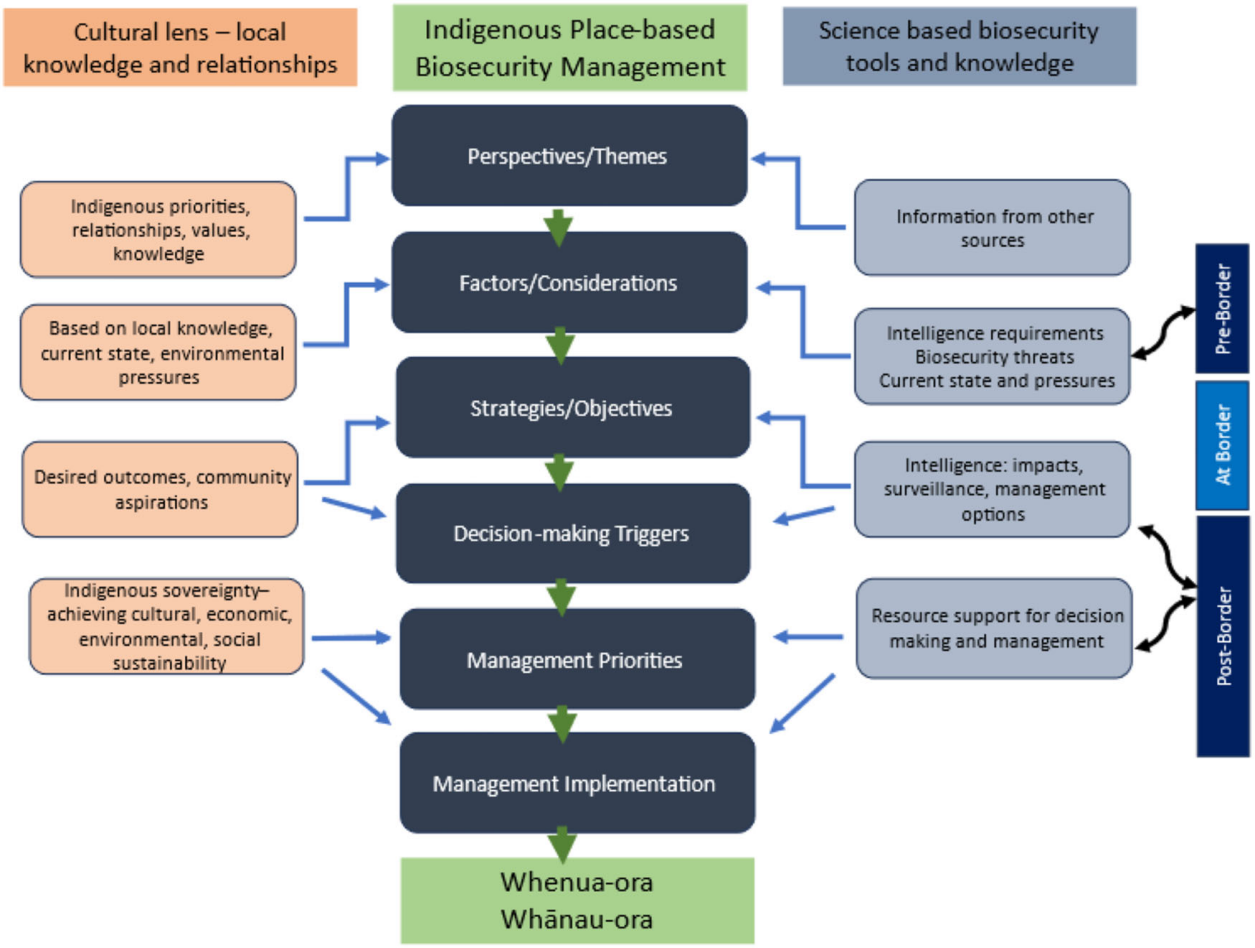
Indigenous place‐based biosecurity management (a dual system of national and locally based decision‐making and management). Centered on place, Indigenous communities lead and inform key actions, which are also informed and supported by requisite biosecurity science and tools. Certain biosecurity science components align with New Zealand's current pre‐ and post‐border biosecurity management.

We propose an alternative place‐based approach for biosecurity management that reflects a multiple‐threat, single‐location model, which is an inverse approach to New Zealand's current post‐border biosecurity management. We also propose extending the inclusion of Indigenous participation to the level of biosecurity decision‐making, thereby increasing Indigenous autonomy in the management of Indigenous lands (Figure 3). Indigenous relationships with place reflect intergenerational empiricism, supporting hypotheses forming for managing natural systems that is culturally important and strengthens environmental management (Kuru et al., 2021).

A multiple‐threat, single‐location model may better address biosecurity threats to native species and ecosystems, considering multiple threats and place‐based factors existing at place. New Zealand's current pre‐border and border biosecurity management is a higher level example of this approach; biosecurity threats are targeted as multiple (potential) hazards to a single location.

Risk and impact assessment informing effective biosecurity management across multiple locations in post‐border scenarios appears problematic because of potentially diverse factors (e.g., differing ecosystem types, climates, and abiotic and biotic factors) among locations. Certain post‐border management components reflect a “multiple‐threat, single‐location” approach at the regional scale, where organizations, such as regional councils, locally based industries, and the Department of Conservation, work regionally to manage non‐native species that cannot be eradicated. A further emphasis on local contexts would create opportunities for Indigenous voices, acknowledging the intimacy of Indigenous relationships with lands and nature in tribal areas.

A reciprocal Indigenous place‐based model for biosecurity management based on multiple threats to a given area provides an opportunity for activating the value of Indigenous inputs to the biosecurity system (Figure 3). Place‐based management is broad based, applicable to wider environmental and conservation settings, and underpinned by a series of actions, each informed by dual streams of science and culture. This centering of place primarily addresses its significance to Indigenous communities. However, place‐based management may also be advantageous from biological and ecological perspectives. In our proposed approach, place‐based perspectives and themes are identified by the community, and science or other knowledges may be used to elucidate themes. Subsequent actions are principally guided by cultural elements and values but are also informed by biosecurity science and tools. The outcome of the proposed model *Whenua‐ora, Whānau‐ora* (healthy land, healthy people) reflects the interconnection between healthy land and healthy communities. As with New Zealand's current biosecurity model, pre‐border, border, and post‐border levels link directly with biosecurity science, although there is potential for applying specific cultural elements at each of these levels.

## DISCUSSION

Implementing an Indigenous place‐based biosecurity model in New Zealand for biosecurity threats to native species and ecosystems, if not for all biosecurity threats, requires significant system adjustments and shared power and decision‐making. Pre‐border and border protections, which are vital system components for an island nation, should remain within a nationally focused and governed framework; however, post‐border protections could be partially devolved to local (with anticipation of tribal) areas. Devolvement would comprise a dual system, resourced and supported nationally but with decision‐making and management occurring locally.

Elements of the proposed system change for New Zealand's biosecurity currently exist in Australia. The Australian system is managed by the federal government, which maintains pre‐border and border protections, although, within Australian borders, management and protection are overseen at the state and territorial levels (DAFF, 2022). The Australian example differs from the approach proposed here because the state and territory boundaries were not delineated with biosecurity risk management in mind, the post‐border biosecurity risk management still reflects the Western paradigm, and the geographical scales of the states and territories are vast. However, it demonstrates the benefits and complexities of a similar biosecurity system devolving biosecurity response and management to regions or territories in the nation‐state.

As island nations, New Zealand and Australia share many biosecurity concerns and similar border protection issues and maintain a strong emphasis on protecting native flora and fauna from biosecurity threats. Both countries are concerned with protecting primary industries and working collaboratively with industry stakeholders. According to the Australian Department of Agriculture, Fisheries and Forestry, the value of Australia's agriculture, forestry, and fisheries export industries is estimated at $51 billion. However, this pales in comparison with the country's environmental assets (estimated value of $5.7 trillion [DAFF, 2022]). Likewise, the export value of New Zealand's primary industries is approximately $57 billion (MPI, 2023). A similar figure for New Zealand's environmental assets could not be ascertained, although, like Australia, this may be higher than primary production exports. The assumed economic value of New Zealand's natural estate, along with the associated risks and impacts of biosecurity threats to native species and forests, significantly supports increasing attention to threats that impact New Zealand's natural environment. In conjunction with the importance of Indigenous relationships with place, this further highlights the need for more effective biosecurity management and consideration of alternatives, such as the proposed model.

An emphasis on place‐based biosecurity management draws parallels with ongoing shifts in conservation and biodiversity management toward incorporating biocultural approaches grounded in place (Barton et al., 2021; Lyver et al., 2019; Morishige et al., 2018). This shift is also evident relative to ecological data, for which issues of Indigenous data sovereignty are being advanced. For example, the Local Contexts Hub is an international initiative offering tools for Indigenous communities to apply or reapply cultural authority over heritage collections and data (Local Contexts, 2023). These tools include traditional knowledge and biocultural labels, digital mechanisms that combined with the Local Contexts Hub enable community control over the use and distribution of their place‐specific data.

Also addressed by the proposed model is the tendency for Indigenous voices in biosecurity management and decision‐making to be confined to cultural values, or perhaps extending to environmental values. Isolating Indigenous perspectives fails to recognize that Indigenous communities do not exist in cultural isolation but are heavily influenced by economic, environmental, and social conditions at local, national, and international scales. It is therefore appropriate that Indigenous perspectives overlay all biosecurity risk categories and not be confined to cultural values. While strengthening Indigenous relationships with their respective lands, place‐based biosecurity management also avoids applying a universal cultural approach and instead encapsulates the plurality of different tribal groups.

With our proposed model, we advocate for addressing biosecurity threats to New Zealand's native biodiversity and landscapes through a paradigm shift from valuing Indigenous participation to recognizing (and including) the value of Indigenous participation in the biosecurity system. This shift reflects a move from non‐Indigenous paradigms to paradigms that elevate Indigenous voices to decision‐making and leadership in biosecurity response and management of Indigenous lands. The enduring familial connections Māori maintain with tribal lands and nature provide intergenerational perspectives that complement the current and more immediate focus of New Zealand's biosecurity system.

Inclusive decision‐making and management are vital to addressing biosecurity, conservation, and environmental issues in a manner that respects diverse communities, their relationships with place, and our shared planet. Notwithstanding progress in advancing Indigenous participation in the environmental sphere (in New Zealand and globally), there remains considerable room for improvement. Shifting paradigms from current Western‐based models to approaches that reflect Indigenous paradigms offers an alternative future for collaborative environmental management.

Our proposed approach provides a pathway for Indigenous biosecurity management that avoids isolation of cultural perspectives and strengthens existing Indigenous relationships with place. Biosecurity science and science‐based tools remain an important component of the approach, supporting the complementary aspects between science and (Indigenous) culture as society navigates humanity's collective future.

## AUTHOR CONTRIBUTIONS

**Tracey Godfery**: Conceptualization; writing—original draft; writing—review and editing. **Nari Williams**: Supervision; writing—review and editing. **John Kean** **Daniel Hikuroa** and **Andrew Robinson**: Writing—review and editing.
